# Sesamol loaded solid lipid nanoparticles: a promising intervention for control of carbon tetrachloride induced hepatotoxicity

**DOI:** 10.1186/s12906-015-0655-y

**Published:** 2015-05-03

**Authors:** Neha Singh, Neeraj Khullar, Vandita Kakkar, Indu Pal Kaur

**Affiliations:** Department of Biotechnology, Panjab University, Chandigarh, 160014 India; Department of Pharmaceutics, University Institute of Pharmaceutical Sciences, Panjab University, Chandigarh, 160014 India

**Keywords:** Sesamol, SLNs, CCl_4_, Oxidative stress, Hepatoprotective

## Abstract

**Background:**

Sesamol, a component of sesame seed oil, exhibited significant antioxidant activity in a battery of *in vitro* and *ex vivo* tests including lipid peroxidation induced in rat liver homogenates. Latter established its potential for hepatoprotection. However, limited oral bioavailability, fast elimination (as conjugates) and tendency towards gastric irritation/toxicity (especially forestomach of rodents) may limit its usefulness. Presently, we packaged sesamol into solid lipid nanoparticles (S-SLNs) to enhance its biopharmaceutical performance and compared the efficacy with that of free sesamol and silymarin, a well established hepatoprotectant, against carbon tetrachloride induced hepatic injury in rats, post induction. A self recovery group in which no treatment was given was used to observe the self-healing capacity of liver.

**Methods:**

S-SLNs prepared by microemulsification method were administered to rats post-treatment with CCl_4_ (1 ml/kg body weight (BW) twice weekly for 2 weeks, followed by 1.5 ml/kg BW twice weekly for the subsequent 2 weeks). Liver damage and recovery on treatment was assessed in terms of histopathology, serum injury markers (alanine aminotransferase, aspartate aminotransferase), oxidative stress markers (lipid peroxidation, superoxide dismutase, and reduced glutathione) and a pro-inflammatory response marker (tumor necrosis factor alpha).

**Result:**

S-SLNs (120.30 nm) at a dose of 8 mg/kg BW showed significantly better hepatoprotection than corresponding dose of free sesamol (FS; p < 0.001). Effects achieved with S-SLNs were comparable with silymarin (SILY), administered at a dose of 25 mg/kg BW. Self recovery group confirmed absence of regenerative capacity of hepatic tissue, post injury.

**Conclusion:**

Use of lipidic nanocarrier system for sesamol improved its efficiency to control hepatic injury. Enhanced effect is probably due to: a) improved oral bioavailability, b) controlled and prolonged effect of entrapped sesamol and iii) reduction in irritation and toxicity, if any, upon oral administration. S-SLNs may be considered as a therapeutic option for hepatic ailments as effectiveness post induction of liver injury, is demonstrated presently.

## Background

Several of the drugs recommended for the treatment of liver diseases tend to show adverse effects on the liver function [1]. As the current available choice of useful drug treatments for hepatic dysfunction is highly limited [1], hence there is a continuous search for safe, efficient and alternative therapeutic options [2]. Oxidative stress, or production of reactive oxygen species (ROS) is known to initiate and regulate the progression of liver disease, independent of the etiologic agent [3]. Thus, phytochemicals with significant antioxidant activity are being explored extensively for the treatment of liver disease.

Carbon tetrachloride (CCl_4_) induced liver injury is the best-characterized animal model of xenobiotic-induced free radical-mediated hepatotoxicity [4]. CCl_4_ metabolism begins with the formation of trichloromethyl free radical, CCl_3_* through the action of the mixed function cytochrome P450 oxygenase system of the endoplasmic reticulum [4,5] which elicits production of reactive oxygen intermediates and causes lipid peroxidation (LPO). Latter is responsible for hepatocellular damage and enhanced production of connective tissue [6,7]. CCl_3_* binds to cellular molecules (nucleic acid, protein, lipid), impairing crucial cellular processes such as lipid metabolism, and resulting in fatty degeneration i.e. steatosis [8]. It can also react with oxygen to form the trichloromethyl peroxy radical CCl_3_OO*, a highly reactive species which initiates the chain reaction of LPO as it attacks and destroys polyunsaturated fatty acids, in particular those associated with phospholipids [8].

Sesamol is a phenol component of sesame oil [9] and is formed by hydrolysis of sesamolin during thermal oxidation [10]. Sesamol, 5-hydroxy-1, 3-benzodioxole or 3,4-methylenedioxyphenol is a well-established antioxidant molecule [11-13]. Its antioxidant activity is attributed to the presence of the benzodioxole group in its ring, which scavenges hydroxyl radical to produce another antioxidant molecule 1,2-dihydroxybenzene [14] that assigns significant hepatoprotective effects to sesamol. Oral bioavailability (BA) of 35.5 ± 8.5% has been reported for sesamol in Sprague Dawley rats [15]. Further, sesamol conjugated metabolites were found to be widely distributed in rat tissues, with the highest concentrations in the liver and kidneys upon oral administration of 100 mg/kg BW in rats, with their rapid elimination via urine and feces within 0–4 h [16]. The Carcinogenic Potency Database indicates that sesamol could be a potential cause of forestomach cancers in rodents with determined TD_50_ values of 1.35 and 4.5 g/kg/day dose in mice and rats respectively [17]. Although the point is not of much concern considering that there are no forestomachs in humans and the TD_50_ dose is much higher than the pharmacological per oral dose of 50–100 mg/kg/day reported thus far, however, the observed effect may however be attributed to its irritant nature due to the phenolic group present in its structure [18].

Sesamol has been reported to possess hepatoprotective properties [19-26] in several models of hepatotoxicity. It may however be noted that sesamol till date has been evaluated in acute model of hepatotoxicity carried for a single dose administration, except for a study wherein sesamol was repetitively administered subcutaneously at a dose of 10 mg/kg, at 0, 6, 12, 18, 24, 30, 36, and 42 h after cecal ligation and puncture, in rats [19]. Sesamol was invariably administered parenterally through subcutaneous route [19,20,23] or intra peritoneal (i.p) route [21,22] in these studies. Further antioxidant effects in terms of reduced glutathione (GSH), alanine aminotransferase (ALT), aspartate aminotransferase (AST) and LPO were observed mainly at doses ≥ 10 mg e.g. 10 mg [19,22], 20 mg [20] and 41 mg [21] respectively. In an early report the protective effects of sesamol and its related compounds on CCl_4_ induced liver injury in rats was studied [26]. Sesamol exhibited significant effect in case of i.p, subcutaneous and oral administration by alleviating ALT, AST, lactate dehydrogenase, alkaline phosphatase, direct bilirubin and total bilirubin parameters. However, the study was an acute study wherein CCl_4_ was administered at a dose of 0.3 ml/kg and treatment with sesamol was given 2 h prior or after. Even at a dose as high as 276.24 mg given orally complete restoration of the elevated parameters was not achieved. Previously we have reported that sesamol could be used for preventive therapy for CCl_4_ induced sub-chronic hepatotoxicity when loaded into a suitable delivery system at a dose of 8 mg/kg [27].

In view of fast elimination, low BA, an irritant nature and absence of extensive sub-chronic/chronic studies evaluating hepatoprotective effects of sesamol, it was presently proposed to explore the therapeutic and hepatoprotective potential of sesamol loaded into solid lipid nanoparticles (SLNs). It is noteworthy that the present study evaluated the ability of bioavailable sesamol (S-SLNs) to provide hepatoprotection post induction of liver injury. This is in sharp contrast to the proposed protective role of these natural agents upon pretreatment. SLNs are an excellent colloidal carrier system [28,29] whose small size (<200 nm) helps to overwhelm the reticuloendothelial system (RES) pick up such that these particles recirculate or pass several times through liver tissues thus achieving an enhanced (both intensity and duration) effect of the entrapped agent [27,30].

A well established hepatoprotective agent silymarin (SILY) [31] was used to compare the efficacy of S-SLNs and free sesamol (FS). Additionally, a self recovery (SR) group which did not undergo any treatment after induction of hepatotoxicity was also included in the study to rule out false implications of the therapy. The objectives of the present study were to evaluate the efficacy of S-SLNs in terms of serum biochemical markers of liver injury i.e. ALT, AST, antioxidant markers, a pro-inflammatory marker i.e. tumor necrosis factor-α (TNF-α) and histopathological investigations, in CCl_4_ induced liver injury in rats. However, only the basic histological, biochemical and molecular studies (due to limitation of animals to be used and funds available) were selected as evaluation parameters because the main objective of the study was not to elicit the mechanism of action of sesamol which is already well illustrated, but to establish the hepatoprotective superiority of the formulated S-SLNs over the free sesamol form– (i) upon oral administration, (ii) at clinically relevant doses, and (iii) as therapeutic agents for chronic liver diseases.

## Methods

### Materials

Sesamol was obtained as a free gift from Jubilant Life Sciences (Noida, Uttar Pradesh, India); Compritol 888 ATO® was a gift sample from Gattefosse, USA. Soy Lecithin (Hi Media, India); Tween 80 (S.D. Fine Chemicals, India) and CCl_4_ (Merck) were also used in the study. All other chemicals and reagents were of analytical grade.

### Preparation of S-SLNs

SLNs were prepared by the microemulsification method, as reported in our previous work [27]. Briefly, polysorbate 80 (45.45%), soy lecithin (0.58%) and water were placed together in a beaker and heated to the lipid melting temperature. Lipid (7.27%) was also melted at 82–85°C separately. Sesamol was added to the aqueous phase containing polysorbate 80, following which the hot aqueous emulsifier mix, was dropped at once into the lipid melt, under magnetic stirring to obtain a clear microemulsion. The hot microemulsion thus formed, was transferred into an equivalent amount of cold water (~2°C) under continuous mechanical stirring (5000 rpm) for 1.5 h. In the aqueous medium, SLNs are formed by crystallization of the hot lipid droplets present in the microemulsions [32]. The prepared aqueous SLN dispersion was stored in a refrigerator until further analysis.

### Characterization of SLNs

The average particle size, polydispersity index (PDI) and zeta potential of SLN dispersion was determined using DelsaNano C, Beckman Coulter, Inc. The morphology of SLNs was examined using an electronic transmission microscope (Hitachi H-100, Japan). The total drug content (TDC) and the % entrapment of sesamol in SLNs were determined as described in our previous work [27,33]. Briefly, TDC was estimated spectrophotometrically at λ_max_ of 294 nm by disrupting 1 mL of the SLN dispersion using an appropriate volume of chloroform: methanol (1:1, v/v). The % entrapment was estimated by dialysis bag method [27,33] where 1 ml of S-SLN dispersion was dialysed against 100 ml of water to remove the free sesamol, maintained at 37°C and stirred at 150 rpm, using dialysis bag (12KDa, Hi Media, India). After 15 min SLN dispersion remaining in the dialysis bag, i.e. the dialysate was disrupted with a suitable quantity of chloroform: methanol (1:1, v/v) and the clear solution was analysed spectrophotometrically to give a direct measure of the entrapped sesamol/ml of the SLN dispersion.

### Animal model and experimental protocol

Male Wistar rats weighing 150 to 200 gm were used in the study. The experimental protocols were approved by the Institutional Animals Ethical Committee of Panjab University, Chandigarh (letter no 5381/VCD 6/12/10, meeting held on 29.11.10). Rats were fed standard chow diet and tap water *ad libitum*. The animals were housed 4 to 5 per cage.

Animals were randomly divided into different groups, each having 6 animals except for the naive control (NC) group and per se (PS) groups which comprised of 3 animals each. In the positive control (CCl_4_) group, hepatic injury was induced by oral administration of 1 ml/kg BW CCl_4_ (mixed with an equal volume of olive oil) twice a week for first two weeks followed by 1.5 ml/kg body weight (BW) for the subsequent two weeks. In the vehicle control (VC) group 1 ml/kg BW olive oil was administered twice a week for first two weeks and 1.5 ml/kg for the subsequent two weeks. In the S-SLN group three days after administering the last dose of CCl_4_ as in the positive control group, S-SLNs were administered at a dose of 8 mg/kg BW daily for four weeks. Similarly, in the FS group, three days post last dose of CCl_4,_ FS dissolved in distilled water was administered orally at a dose of 8 mg/kg BW daily for four weeks. In SILY group three days post last dose of CCl_4_, SILY (dispersed in 0.7% w/v carboxy methyl cellulose in water) was administered at a dose of 25 mg/kg BW [34] daily for four weeks. In the SR group after the final dose of CCl_4_ was administered, rats were left for self recovery for four weeks. In the NC group the animals were kept on normal diet throughout the eight week period of study. In the S-SLNs PS group after four weeks of normal diet S-SLNs were administered to rats at a dose equivalent of 8 mg of sesamol/kg BW daily for four weeks. Similarly, in the FS PS, after four weeks of normal diet, FS dissolved in distilled water was administered orally at a dose of 8 mg/kg BW daily for four weeks. In blank-SLNs per se group (Bla-SLNs PS) blank SLNs were administered orally daily for four weeks after rats were kept at normal diet for four weeks. In the CCl_4_ group animals were sacrificed three days post final dose of CCl_4_ i.e. on 32^nd^ day and all the other animals were sacrificed on the 60th day, i.e. a day after the final treatment schedule (no treatment in the case of NC, VC and SR groups).

Blood sampling was done via retro-orbital plexus, in ether anesthetized rats. Serum was isolated by centrifuging blood samples at 4°C for 20 min at 4000 rpm and was stored at −20°C. Rats were sacrificed by cervical dislocation and their livers were harvested after the end of each study. A part of the liver was stored at 10% buffered formalin for histopathological examinations and the remaining part was homogenized with 10% (w/v) cold phosphate –buffered saline (PBS 0.1Mol/l, pH 7.4). The oxidative stress parameters were determined in the liver post –mitochondrial supernatant (PMS) prepared by centrifuging rat liver homogenates in the chilled phosphate buffer, pH 7.4 at 10,500 g for 20 min at 4°C. The following tests were performed to assess the therapeutic potential of S-SLNs and other groups under study.

### Histological examinations of liver

Representative liver tissue of different groups was processed, stained with haematoxylin & eosin (HE) and examined under the light microscope for steatosis, inflammation, necrosis, fibrosis and cirrhosis. The histological changes were observed on HE stained sections [27,35]. Changes in the histological parameters for liver tissue, i.e. fatty changes, inflammation and fibrosis were graded as follows: (−) showing no occurrence and +, ++, +++ and ++++ as mild, medium, high and severe occurrences respectively.

### Estimation of serum liver injury markers

Serum of animals of the respective groups stored at – 20°C was used to measure ALT and AST levels according to Reitman & Frankel’s method [36]. For ALT estimation 0.5 ml of the substrate comprising 0.2 M alanine, 2.0 mM ketoglutarate and 100 mM phosphate buffer, pH 7.4, was taken. However, the substrate for AST comprised of 0.2 M aspartate, 2.0 mM ketoglutarate and 100 mM phosphate buffer, pH 7.4. Both serum AST & ALT was expressed as nmoles/nL and was compared with sodium pyruvate as the standard.

### Estimation of antioxidant parameters

The oxidative stress parameters were determined in liver PMS.

#### Estimation of LPO

The quantitative measurement of LPO in liver was done according to the method of Wills [37]. The malondialdehyde (MDA) content, a measure of LPO, was assayed as liver thiobarbituric acid reactive substances. The results were expressed as nanomoles of MDA per milligram of protein, using the molar extinction coefficient of the chromophore as 1.56 × 105 M ^− 1^ cm ^− 1^.

#### Measurement of superoxide dismutase (SOD)

SOD activity was assayed according to the method of Kono *et al.* [38]. SOD activity was expressed in terms of units of SOD per milligram of protein (SOD units/mg Pr).

#### Estimation of GSH levels

GSH was estimated by the method of Jollow *et a*l. [39]. The results were expressed as nmoles of GSH per μg of protein (nmoles of GSH/μg Pr).

#### Estimation of pro-inflammatory cytokine, TNF-α

TNF-α levels were measured in liver homogenates using an ELISA kit (RayBiotech, Inc). The assay was performed according to the manufacturer protocol.

### Statistical Analysis

Data expressed as mean ± standard deviation (SD), were analysed through one way analysis of variance (ANOVA), followed by the Tukey test for comparison of various treatments using Sigma stat 3.5. Differences were considered statistically significant at p ≤ 0.001 or p < 0.05 as indicated suitably.

## Results

### Characterization of SLNs

**Figure 1:**
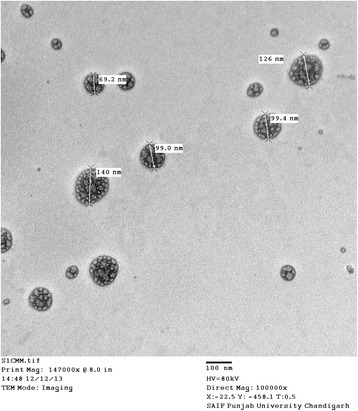
TEM micrograph of S-SLNS.

The average particle size, PDI and zeta potential of S-SLNs was found to be 120.30 nm, 0.111 and −51.31 mV respectively. When observed under TEM, S-SLNs were found to be spherical in shape (Figure 1). The TDC and % entrapment of S-SLNs were estimated to be 3.31 ± 0.01 mg/ml and 73.92 ± 2.49% respectively (n = 3).

### Histological analysis

**Figure 2:**
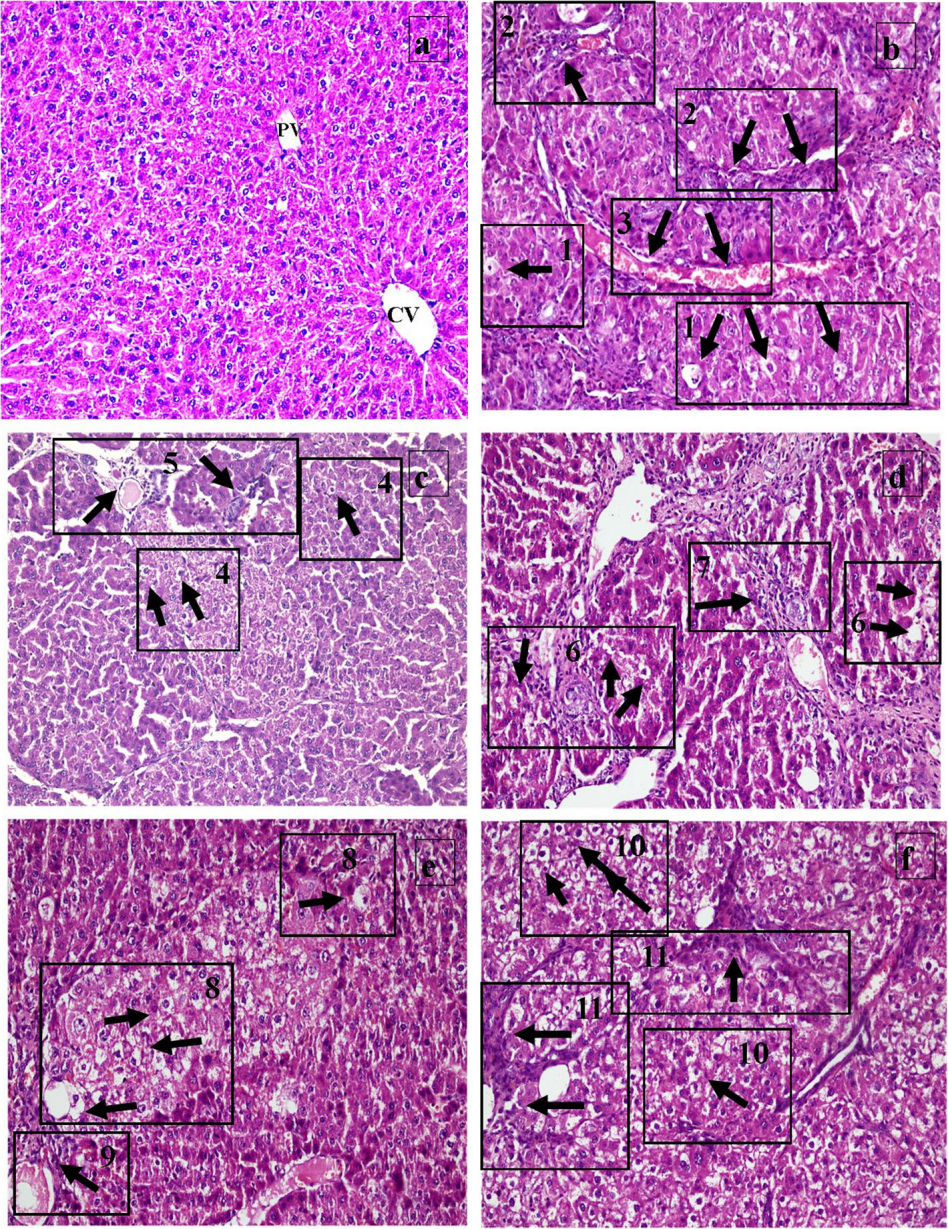
Haemotoxylin and eosin (HE) stained liver section of vehicle control animals showing polyhedral hepatocytes (H), central vein (CV) and portal vein (PV) (2a); rats administered CCl_4_ showing altered histoarchitecture characterized by fatty changes i.e. steatosis [1], inflammatory bridges [2] and vascular bridges [3] 2(b); S-SLNs treated rats showing mild steatosis [4] and medium portal inflammation [5] 2(c); FS treated rats showing medium steatosis [6], high inflammation [7]; SILY treated rats showing medium steatosis [8] and inflammation [9] 2(e); and untreated SR group showing altered histoarchitecture with high occurrence of steatosis [10] and inflammation [11] 2(f). Representative views of each group are presented at 200X magnification.

The hepatoprotective effects of S-SLNs were evaluated by histological analysis. Representative views of liver sections are shown in Figure 2 (a-f). Figure 2a represents the VC liver sections stained with HE showing normal histoarchitecture. The PS and NC groups showed features similar to VC group and hence are not shown. However, liver tissue in the rats treated with CCl_4_ revealed extensive liver injuries, characterized by fatty cells, inflammatory bridges and vascular bridges (Figure 2b). Howsoever, treatment with S-SLNs (Figure 2c) improved the state of steatosis, ameliorated inflammation as compared to CCl_4_ group. In comparison, FS treatment did not restore the histoarchitecture as indicated by the presence of steatosis, and dense portal inflammation (Figure 2d). SILY group, also showed a significant improvement, with medium steatosis and portal inflammation being evident in the observed histological sections (Figure 2e) which were comparable to the S-SLNs group. The SR group showed (Figure 2f) altered histoarchitecture with high occurrence of steatosis and inflammation indicating that the liver is unable to restore its architecture in the absence of any treatment. The obtained results are in terms of the extent of hepatic injury and compiled in Table 1.

**Table 1:** **Histological grading of CCl**_**4**_ **induced hepatic injury in male Wistar rats**

**Group**	**Fatty changes**	**Inflammation**	**Fibrosis**
**NC**	**-**	**-**	**-**
**VC**	**-**	**-**	**-**
**S-SLNs PS**	**-**	**-**	**-**
**FS PS**	**-**	**-**	**-**
**Bla SLNs PS**	**-**	**-**	**-**
**CCl**_**4**_	**++++**	**++++**	**++++**
**S-SLNs**	**+**	**++**	**++**
**FS**	**++**	**+++**	**+++**
**SILY**	**++**	**++**	**++**
**SR**	**+++**	**+++**	**++++**

-, normal occurrence; +, mild occurrence; ++, medium occurrence; +++, high occurrence; ++++, severe occurrence.

### Serum liver injury markers

Administration of CCl_4_ led to 2570.07% increase in the ALT levels as compared to the VC group. However, S-SLNs and FS treatment significantly decreased the elevated ALT levels by 52.59 ± 2.37% and 34.12 ± 3.69% respectively. Results obtained with S-SLNs were significantly better (p < 0.001) than CCl_4,_ FS and SR groups. The effect was, however comparable to SILY (as shown in Table 2), though the latter was administered at a 3 times higher dose of 25 mg/kg BW. Similarly, AST levels were increased by 890.89% in the CCl_4_ group with respect to the VC group and the level significantly decreased in S-SLNs (p < 0.001) treated group as compared to the CCl_4_, FS and SR groups. S-SLNs attenuated the increased AST levels by 75.68 ± 3.61%. The latter may be expressed as a 4.13 times improvement as compared to a 2.09 times improvement observed in the FS group.

**Table 2:** **% inhibition in ALT and AST levels with respect to CCl**_**4**_ **group, % increase in ALT and AST levels with respect to VC group and alleviation of the hepatic damage in terms of fold reduction in increased enzyme levels change with respect to CCl**_**4**_

		**ALT Levels**		**AST Levels**	
**Groups**	**Dose (mg/kg)**	**% Inhibition versus CCl**_**4**_ **group (% increase with respect to VC)**	**Alleviation (Fold change with respect to CCl**_**4**_ **)**	**% Inhibition versus CCl**_**4**_ **group (% increase with respect to VC)**	**Alleviation (Fold change with respect to CCl**_**4**_ **)**
S-SLNs^a^	8	52.59 ± 2.37 (1073.93 ± 166.43)	2.12	75.68 ± 3.61 (139.52 ± 51.44)	4.13
FS	8	34.12 ± 3.69 (1677.97 ± 298.08)	1.51	51.84 ± 5.88 (356.79 ± 53.86)	2.09
SILY^a^	25	48.84 ± 3.15 (1173.98 ± 166.43)	1.95	75.97 ± 4.04 (129.95 ± 31.47)	4.18
SR	-	19.09 ± 1.38 (1677.97 ± 298.08)	1.23	5.22 ± 2.22 (837.98 ± 148.87)	1.05

Data are expressed as mean ± S.D (n = 6) except for fold change. ^a^All the groups are significantly different from each other at p ≤ 0.001 except for those marked similarly. CCl_4_ increased the ALT and AST levels by 2570.07% and 890.89% respectively with respect to VC. Actual values for CCl_4_ and VC for ALT are 7.21 ± 0.35 and 0.27 ± 0.05 respectively; AST are 6.50 ± 0.61 and 0.93 ± 0.20 respectively. The actual values are expressed in units of nmoles/nL.

### Antioxidant parameters

#### LPO

**Figure 3:**
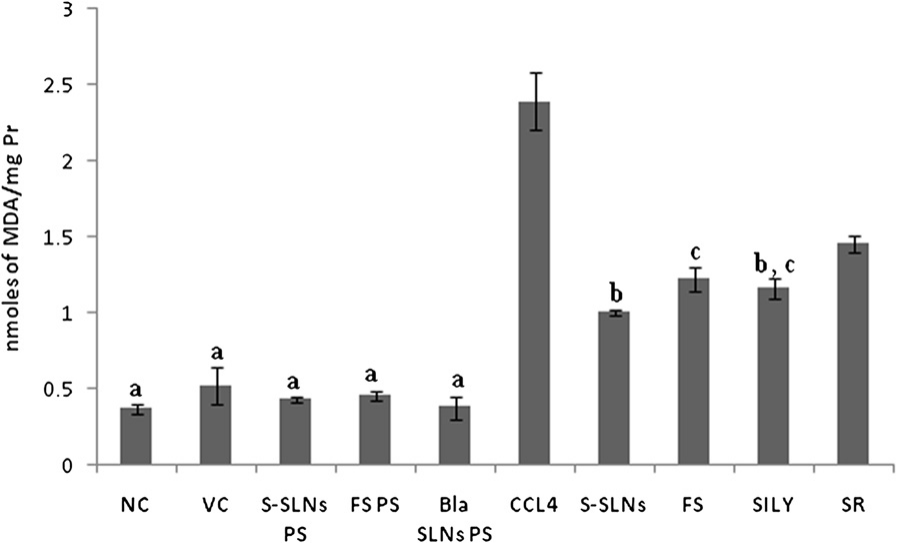
MDA levels after treatment with S-SLNs, FS, SILY and various other control groups post CCl_4_ induced hepatic injury. Data are expressed as mean ± S.D (n = 6); except NC and PS groups (n = 3). All groups are significantly different from each other at p < 0.001 or p < 0.050 except for those marked similarly.

LPO expressed as MDA levels increased significantly (p < 0.001) in CCl_4_ group as compared to NC and VC group. There was attenuation in LPO as indicated by a significant decrease in levels of MDA in S-SLNs (p < 0.001) group with respect to CCl_4_ group. S-SLNs treatment was significantly more effective (2.38 fold) in reducing (p < 0.001) MDA levels as compared to SR (1.64 fold) group and FS (1.96 fold; p < 0.05) group. The attenuation was comparable to SILY group (2.04 fold). There was no significant difference in the values of NC, VC and PS groups (Figure 3).

#### SOD levels

**Figure 4:**
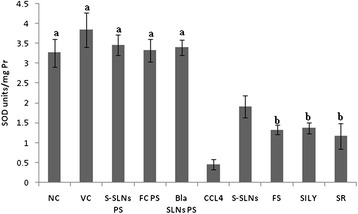
SOD levels following treatment with S-SLNs, FS, SILY and various other control groups post CCl_4_ induced hepatic injury. Data are expressed as mean ± S.D (n = 6); except NC and PS groups (n = 3). All groups are significantly different from each other at p < 0.001 or p < 0.050 except for those marked similarly.

SOD levels were found to be significantly (p < 0.001) low in CCl_4_ group as compared to NC and VC group. Treatment with S-SLNs however, increased the SOD levels (4.19 times) significantly (p < 0.05) as compared to FS (2.91 times) and SILY (3.01 times) groups. The SR group showed 2.56 times improvement in SOD levels (Figure 4), however the effects achieved in the S-SLNs were significantly better (p = 0.001) than SR group. There was no significant difference in the values of NC, VC and PS groups.

#### GSH levels

**Figure 5:**
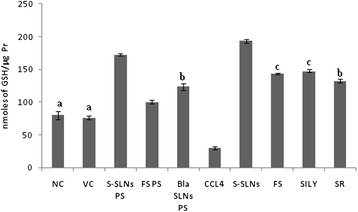
GSH levels after treatment with S-SLNs, FS, SILY and various other control groups post CCl_4_ induced hepatic injury. Data are expressed as mean ± S.D (n = 6); except NC and PS groups (n = 3). All groups are significantly different from each other at p < 0.001 or p < 0.050 except for those marked similarly.

The level of total GSH was significantly reduced (p < 0.001) by CCl_4_ as compared to the VC and NC group. Treatment with S-SLNs countered the inhibitory effect of CCl_4_ by significantly (p < 0.001) increasing (6.49 times) the GSH levels as compared to FS (4.81 times), SILY (4.94 times) and SR (4.44 times) groups. Furthermore, Bla-SLNs PS and S-SLNs PS groups very interestingly showed a significant increase in the GSH levels in comparison to NC and VC groups (Figure 5).

### Inflammatory response

**Figure 6:**
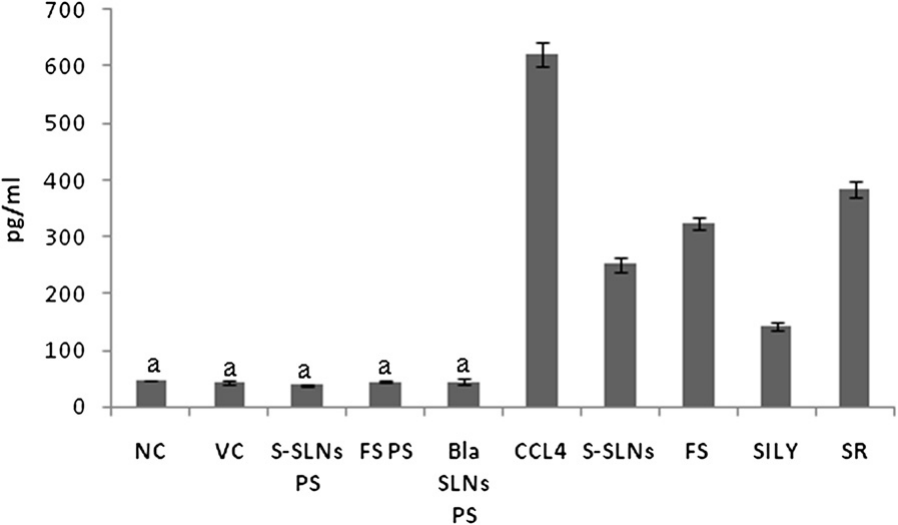
TNF-α levels after treatment with S-SLNs, FS, SILY and various other control groups post CCl_4_ induced hepatic injury. Data are expressed as mean ± S.D (n = 6); except NC and PS groups (n = 3). All groups are significantly different from each other at p < 0.001 except for those marked similarly.

The levels of the proinflammatory cytokine TNF-α were determined in the liver. As shown in Figure 6, compared with the VC group, level of TNF-α was found to be significantly elevated in the CCl_4_ (1379.76 ± 128.85%) group. S-SLN treatment significantly reduced (2.47 times) the elevated levels of TNF-α (p < 0.001) as compared to FS (1.91 times) and SR groups (1.62 times). However, SILY group showed vast improvement (4.34 times) which was higher than all other treatment groups. There was no significant difference in the values of NC, VC and PS groups.

## Discussion

The present investigation was an endeavor to explore hepatoprotective effects of S-SLNs in comparison to free drug and silymarin and establish S-SLNs as a therapeutic option for hepatic damage post its induction. Scarce data [40] including couple from our laboratory [27,30] on the significant usefulness of phytochemical loaded SLNs over free drugs is available on the utility of SLNs for liver disorders.

S-SLNs with total drug content of 3.31 ± 0.01 mg/ml and % entrapment of 73.92 ± 2.49% (n = 3), with an average particle size and zeta potential of 120.30 nm (PDI- 0.111) and −51.31 mV, respectively, were prepared by the microemulsification method. The method of preparation and the formula have been optimized in our laboratory [18,33]. It may also be highlighted here that the method of preparation and the formula optimized in our laboratory [28,33] uses dilution of hot microemulsion with a significantly small (1:1) quantity of cold water (usual recommended volume is 1:25 to 1:100) such that it results in a concentrated dispersion. Latter ensures (i) a higher drug content/volume of the dispersion such that suitable doses can be administered in smaller volumes and, (ii) overcomes the need to concentrate the dispersion by dialysis or lyophilisation. Both of which are time consuming and costly techniques.

Size of nanoparticles monitors their uptake, into pathological and inflamed tissues by macrophages, or delivery across the fenestrae of the liver sinusoid [41,42]. It has been reported that nanoparticles with a diameter of less than 200 nm reach liver parenchymal cells and generate significant effects [43,44]. Presence of tween 80 as a surfactant in these SLNs gives them an added advantage of hydrophilicity similar to that imparted by PEGylation [27,30,44]. Nanoparticles having zeta potential > ± 25 mV are stable dispersions with little or no chance of aggregation. Furthermore, a PDI of <0.3 confirms uniform distribution of nanosized particles in the SLN dispersion with low or no incidence of micrometer particles. The latter may also indirectly be taken to confirm that all particles are of nano size and there are no aggregates. The small size of the stable and uniform S-SLN dispersion is expected to result in cumulative uptake by the pathological liver over prolonged period due to repetitive filtration and passes through a probable facilitated transport across the fenestrae of the liver sinusoids. He *et al.* [40] have reported 2.79 times improved oral BA of SILY upon incorporation into SLNs (170.7 nm) which could result in a better drug targeting to the liver.

**Figure 7:**
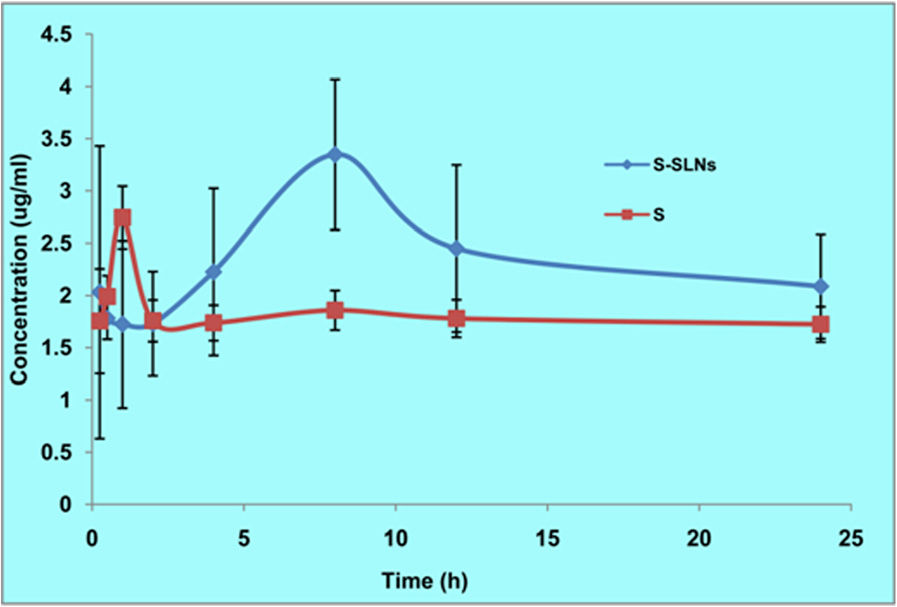
Plasma concentration-time profiles of S-SLNs and FS when administered orally at a dose of 4 mg/kg to mice.* S here stands for free sesamol i.e. FS.

Previously we have reported that drug released versus time data for S-SLNs wherein the total amount of the drug released upto 24 h is 69.8% while a fast release of 28.4% in initial 1 h indicated the burst release of the drug either from the small sized nanoparticles or the release of the unentrapped drug [27]. Further, a controlled release pattern was depicted during 6–11 h, where no significant increase in the % drug released was observed [27]. In the oral pharmacokinetics conducted by us (Figure 7), in a separate study in mice, at a dose of 4 mg/kg BW, administered S-SLNs indicated a 1.32 times better (p < 0.05) BA (AUC_0-∞_) than FS (unpublished work). It may however be noted that the study was performed in mice and at a lower dose of 4 mg/kg BW (8 mg/kg BW dose of sesamol was administered to rats presently) and since the latter are usually fast metabolisers so the effect achieved with S-SLNs in rats and also in humans, may actually be more pronounced. Further, a higher t_max_ (8 h) value observed for S-SLNs, in comparison to 1 h for FS, indicates a sustained effect, probably achieved due to slow release of the drug from the lipidic nanoparticles (unpublished work).

Presently, CCl_4_ administration lead to hepatic steatosis, inflammation, and the formation of the vascular and fibrotic bridges between nodules as revealed by the histological studies and these observations were similar to previous reported studies [27,35]. Present investigations revealed improvement in histoarchitecture of liver on treatment with S-SLNs which was significantly better than FS and SR groups and comparable to the SILY group. In an earlier report sesamol exhibited renoprotective effects in streptozotocin (STZ) induced diabetes in rats [45] as it modulated the release of profibrotic cytokines i.e. tissue growth factor-1 beta (TGF-β1). Similar effect in the present study may have led to reduction in the fibrotic bridges in S-SLNs treated group and better effects observed in comparison with FS and SR groups.

Administration of CCl_4_ to rats lead to a significant elevation (p *<* 0.001) in the levels of serum liver injury markers, i.e. ALT and AST in comparison with the untreated VC and NC group. Increase in AST and ALT levels is attributed to the damaged structural integrity of the liver, as these enzymes are cytoplasmic in location and hence are released into the plasma due to the liver cell damage [46]. Treatment with S-SLNs significantly reduced the serum ALT and AST levels, which were significantly better than FS group and was comparable to SILY (as shown in Table 2), though the latter was administered at a 3 times higher dose of 25 mg/kg BW. Sesamol has exhibited anti-MMP-9 (matrix metallopeptidase 9) activity against monocrotaline-induced SOS in rats [14]. MMP-9 is important for the breakdown and necrosis of hepatocytes [47] resulting in the leakage of ALT and AST from liver. Hence, it may indirectly indicate that the anti-MMP-9 effect of sesamol [23] may protect against hepatic tissue necrosis, by attenuating the increased levels of ALT and AST.

S-SLNs treatment may lead to membrane stabilization of hepatocytes which is indicated presently by the reduction of LPO. CCl_4_ hepatotoxicity is attributed to LPO and increased MDA levels [48] in liver tissue. Administration of S-SLNs significantly reduced (p < 0.001) the CCl_4_ induced increase in MDA levels and the effect was better (p < 0.05) than FS group. This may be assigned to the higher cellular permeability of sesamol achieved by S- SLNs [49]. S-SLNs with a polysorbate 80 and a phospholipid coat of surfactant and co-surfactant respectively, are expected to intermingle with the membrane lipids of liver cells resulting in physiologically significant effects as shown in the present study.

A decreased SOD activity observed in the CCl_4_ treated group was attributable to its easy inactivation by lipid peroxides or ROS. SOD is a sensitive index for the hepatocellular damage [50]. Since treatment with S-SLNs significantly lowered the levels of MDA or ROS, hence, as expected SOD was significantly (p < 0.05) upregulated in S-SLNs group. It is important to note here that S-SLNs treatment was not only significantly more effective than FS but also in comparison to a well established hepatoprotective and antioxidant SILY, presently in clinical use too [51].

GSH is a predominant low-molecular-weight thiol and the most important nonenzyme antioxidant in mammalian cells [52]. It effectively protects cells against oxidative stress caused damage by scavenging free radicals, removing hydrogen peroxide (H_2_O_2_), and suppressing LPO [53]. In our study, treatment with S-SLNs increased the levels of GSH significantly (p < 0.001) as compared to all other groups. Further, even the S-SLNs PS and Bla SLNs PS groups showed an increase in the levels of GSH as compared to NC and VC groups. These results indicate that the SLNs have a unique ability to increase the levels of GSH in vivo*.* These results are similar to those observed and reported by us previously [18,27]. Our formulation contains soy lecithin (a type of phospholipid component of biological cell membranes) which consists of 21% posphatidylcholine, and was administered daily for a time period of four weeks, and could probably lead to an increase in the intrinsic levels of GSH in hepatic tissue. Intake of phosphatidylcholine for a long duration is reported to result in an increase in glutathione levels [54]. Even though the % of soya lecithin in our formulation is very small (0.58%) but since it is now incorporated into a highly bioavailable form, i.e. SLNs, the effect may be observed even at low concentrations.

Hepatic injury is associated with elevated TNF-α level as observed presently in the CCl_4_ group and also reported elsewhere [55]. TNF-α levels are elevated both in the infiltrating inflammatory cells and hepatocytes in chronic liver injuries, including viral or alcoholic liver diseases, hepatitis, ischemia, and biliary obstruction [56]. Exposure to hepatotoxic chemicals facilitates TNF-α to induce necrotic cell death and hepatic apoptosis [57]. Sesamol attenuated TNF-α levels in STZ induced diabetes in rats [45] and in our study also elevated levels of TNF- α were significantly attenuated (p < 0.001) by S-SLN treatment and the effect was significantly (p < 0.001) better than FS group.

## Conclusion

S-SLNs treatment was not only significantly more effective than FS but, also showed effects comparable to a well-established hepatoprotective and antioxidant agent SILY. It may however be noted here that the recommended and presently used dose of SILY (25 mg/kg) is >3 times that used for S-SLNs (8 mg/kg). Hence it may be indirectly concluded that S-SLNs are a significantly better therapeutic than SILY. Significant research is now focused on establishing the multiple biological roles of antioxidant phytochemicals. However, in most of these cases, the effects remain confined to the in vitro laboratory experiments due to lack of translation of their antioxidant activity to physiological systems. Major limitations to the latter are poor solubility, stability (proneness to oxidative and photodegradation), permeability, and bioavailability coupled with a fast metabolism of these molecules. Furthermore the in vitro or preclinical doses at which the effects are obtained are not translatable to human use. Hence their biopharmaceutical enhancement is a must if they have to ‘see the light’ of being clinically relevant. Although solubility and BA of sesamol are significant, however its fast metabolism and subsequent elimination indicates need to package it into a sustained/ prolonged release carrier system to improve its T_1/2_ and hence mean residence time in the body to elicit considerable physiological effect. It may be noted that earlier reported data uses higher doses and parenteral administration of sesamol. Presently an orally administered formulation of sesamol is reported. Furthermore the Bla-SLNs PS groups confirm the absence of any adverse effects and safety of the developed formulation. The study established that sesamol could be used as a therapeutic agent for the treatment of liver diseases provided it is loaded into a suitable delivery system.

## Abbreviations

ALT: Alanine aminotransferase
AST: Aspartate aminotransferase
BA: Bioavailability
BW: Body weight
Bla-SLNs: Blank-solid lipid nanoparticles
CCl4: Carbon tetrachloride
DTNB: 5-5′ dithiobis-2-2 nitrobenzoic acid
FS: Free sesamol
GSH: Reduced glutathione
HE: Haemotoxylin and eosin
LPO: Lipid peroxidation
MDA: Malondialdehyde
NC: Naïve control
PMS: Post –mitochondrial supernatant
PS: Per se
PDI: Polydispersity index
ROS: Reactive oxygen species
S-SLNs: Sesamol loaded solid lipid nanoparticles
SR: Self recovery
SLNs: Solid lipid nanoparticles
SOD: Superoxide dismutase
SILY: Silymarin
TDC: Total drug content
VC: Vehicle control
